# Sesquiterpene and Acetogenin Derivatives from the Marine Red Alga *Laurencia okamurai*

**DOI:** 10.3390/md10122817

**Published:** 2012-12-14

**Authors:** Yi Liang, Xiao-Ming Li, Chuan-Ming Cui, Chun-Shun Li, Hong Sun, Bin-Gui Wang

**Affiliations:** Key Laboratory of Experimental Marine Biology, Institute of Oceanology, Chinese Academy of Sciences, Nanhai Road 7, Qingdao 266071, China; E-Mails: liangyi1984@126.com (Y.L.); lixmqd@yahoo.com.cn (X.-M.L.); chuanming-cui@163.com (C.-M.C.); lichunshun@ms.qdio.ac.cn (C.-S.L.); sunhonghappy@yahoo.cn (H.S.)

**Keywords:** marine alga, *Laurencia okamurai*, bisabolane sesquiterpene, C_12_-acetogenin, brine shrimp lethality

## Abstract

In addition to 13 known compounds, four new bisabolane sesquiterpenes, okamurenes A–D (**1**–**4**), a new chamigrane derivative, okamurene E (**5**), and a new C_12_-acetogenin, okamuragenin (**6**), were isolated from the marine red alga *Laurencia okamurai*. The structures of these compounds were determined through detailed spectroscopic analyses. Of these, okamurenes A and B (**1** and **2**) are the first examples of bromobisabolane sesquiterpenes possessing a phenyl moiety among *Laurencia*-derived sesquiterpenes, while okamuragenin (**6**) was the first acetogenin aldehyde possessing a C_12_-carbon skeleton. Each of the isolated compounds was evaluated for the brine shrimp (*Artemia salina*) lethal assay and 7-hydroxylaurene displayed potent lethality with LD_50_ 1.8 μM.

## 1. Introduction

**Figure 1:**
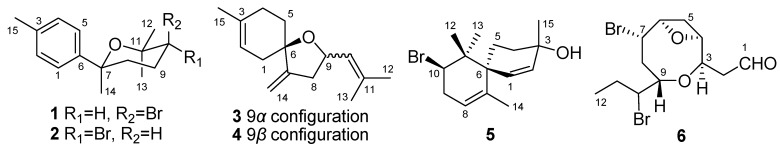
Structures of the isolated new compounds **1**–**6** from *L. okamurai*.

Marine red algae of the genus *Laurencia* are prolific sources of diversified secondary metabolites, predominantly sesquiterpenoids, diterpenoids, and nonterpenoid C_15_-acetogenins [1]. The red alga *Laurencia okamurai*, widely distributed along the coast of China, mainly yields sesquiterpenes and C_15_-acetogenins [2]. These compounds, with structurally diverse skeletons, have attracted much attention for total syntheses [3] as well as chemotaxonomic research [4,5,6]. In the past five years, we have systematically conducted chemical investigation towards eight *Laurencia* species, which have resulted in the isolation of more than 30 new compounds [2,7,8,9,10,11]. In the course of our phytochemical studies on *Laurencia okamurai*, a new, rearranged chamigrane sesquiterpene, laurenokamurin, was previously characterized [10]. Continuous effort on the chemical investigation of this algal species collected from Weihai coastline resulted in the isolation and identification of five new sesquiterpenes, okamurenes A–E (**1**–**5**), one new C_12_-acetogenin, okamuragenin (**6**) (Figure 1), as well as nine known sesquiterpenes and four known C_15_-acetogenins. We present herein the isolation, structure elucidation, and bioactivity of these compounds.

## 2. Results and Discussion

### Structure Elucidation of the New Compounds

Okamurene A (**1**) was obtained as a colorless oil and its molecular formula was established by HRESIMS to be C_15_H_21_BrO, corresponding to five degrees of unsaturation. The ^1^H NMR spectrum of **1** (Table 1) exhibited resonances for a *para*-substituted phenyl unit, four methyl groups, and a brominated or oxygenated methine group. There were also four signals for two diastereotopic methylene protons. The ^13^C NMR and DEPT spectroscopic data (Table 1) revealed the presence of 15 carbon signals including six aromatic carbons (corresponding to a *para*-substituted phenyl unit) and nine aliphatic carbons (corresponding to four methyls, two methylenes, one brominated methine, and two oxygenated quaternary carbons). These units accounted for 4 degrees of unsaturation, requiring one additional ring to be present in **1**.

**Table 1:** ^1^H- and ^13^C-NMR data of compounds **1** and **2** in CDCl_3_^ a^.

No.	1 (CDCl_3_)		2	
	*δ*_H_ (*J* in Hz)	*δ*_C_	*δ*_H_ (*J* in Hz)	*δ*_C_
1/5	7.34, d (8.0)	124.8, CH	7.34, d (8.1)	126.0, CH
2/4	7.11, d (8.0)	128.7, CH	7.12, d (8.1)	128.6, CH
3		136.1, C		136.4, C
6		146.0, C		143.2, C
7		74.6, C		74.4, C
8_eq_	2.16, m	34.1, CH_2_	2.56, m	36.0, CH_2_
8_ax_	2.10, m		2.18, m	
9_eq_	2.28, m	28.2, CH_2_	2.27, m	29.4, CH_2_
9_ax_	2.25, m		1.82, m	
10	4.05, dd (7.9, 4.4)	59.1, CH	4.04, dd (12.1, 4.1)	59.0, CH
11		75.2, C		76.4, C
12	1.47, s	27.8, CH_3_	1.35, s	22.5, CH_3_
13	1.14, s	29.4, CH_3_	0.78, s	30.8, CH_3_
14	1.50, s	31.8, CH_3_	1.36, s	35.8, CH_3_
15	2.23, s	20.9, CH_3_	2.34, s	21.0, CH_3_

^a^ Measured at 500 MHz for ^1^H and 125 MHz for ^13^C.

**Figure 2:**
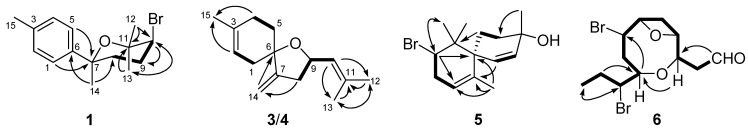
Key COSY (bold lines) and HMBC (arrows) correlations for compounds **1**, **3**/**4**, **5**, and **6**.

The structure of the non-phenyl portion of **1 **was determined by analysis of 2D NMR data (^1^H–^1^H COSY, HSQC, and HMBC). The ^1^H–^1^H COSY experiment established the connectivity for a –CH_2_–CH_2_–CH– unit (C-8 through C-10, Figure 2). The C-10 methine of this unit was connected to CH_3_-12 and CH_3_-13 via the oxygenated quaternary carbon C-11 (*δ*_C_ 75.2) as evidenced by the observed HMBC correlations from the methyl protons H_3_-12 and H_3_-13 to C-10 and C-11, while the C-8 methylene was linked to the CH_3_-14 via the oxygenated quaternary carbon C-7 (*δ*_C_ 74.6) as supported by the observed HMBC correlation from the methyl protons H_3_-14 to C-8 (Figure 2). Given the fact that only one oxygen atom existed in the structure, the linkage of C-7/O/C-11 could be constructed, leading to the formation of a tetrahydropyran moiety, which accounted for the remaining degree of unsaturation. Thus, the planar structure of **1** was assigned.

**Figure 3:**
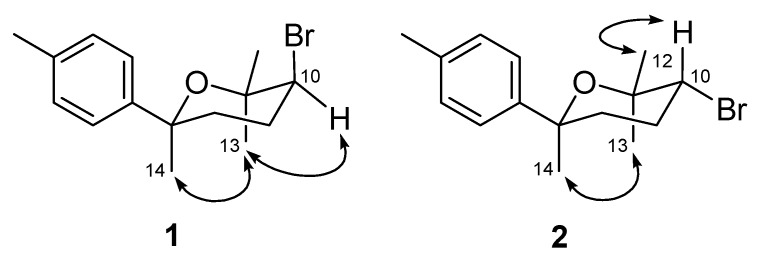
Key NOESY correlations for compounds **1 **and **2**.

Analysis of the proton coupling constants and NOESY data enabled assignment of the relative configuration of **1**. The appearance of the bromomethine proton H-10 as a double doublet, with coupling constants of 7.9 and 4.4 Hz, suggesting the equatorial orientation of H-10 for **1**. In the NOESY spectrum, NOE correlations of H_3_-13 with both H-10 and H_3_-14 placed the methyl groups CH_3_-13 and CH_3_-14 on the same face (axial or pseudoaxial) of the tetrahydropyran ring (Figure 3). On the basis of the above evidence, the structure of **1** was determined, and the trivial name okamurene A was assigned.

The ^1^H and ^13^C NMR spectroscopic data of okamurene B (**2**), an isomer of **1** as established by HRESIMS data, were very similar to those of **1** except for some chemical shift variations of signals corresponding to the C-8, C-9, and C-12 through C-14 (Table 1). Therefore, compound **2** was presumed to be a stereoisomer of **1**. Detailed analysis of the ^1^H and ^13^C NMR data as well as ^1^H–^1^H COSY and HMBC correlations supported the conclusion that **2** possesses the same planar structure as **1**. However, comparisons of the *J*-value and NOESY data of **2** with those of **1** revealed a difference in relative configuration at C-10. A *trans*-diaxial *J*-value for H_ax_-10 and H_ax_-9 (12.1 Hz) indicated an equatorial orientation for the Br-atom at C-10. The NOE correlation from H-10 to H_3_-12 in the NOESY spectrum indicated an equatorial face of CH_3_-12, while the NOE correlation from H_3_-13 to H_3_-14 placed these two methyl groups in axial orientation (Figure 3). Based on the above data, the structure of compound **2 **was identified and it was named okamurene B.

Okamurenes C (**3**) and D (**4**) were obtained as a colorless oily mixture in a 2:1 ratio, as indicated by the ^1^H NMR spectrum. Attempts to separate the mixture by various CC steps using different solvent systems failed. On the other hand, there is no conjugated system in compounds **3** and **4**, making these compounds unsuitable for HPLC separation using the available UV detector. A similar unseparable mixture containing (9*S*)- and (9*R*)-2-bromo-3-chloro-6,9-epoxybisabola-7(14),10-diene from *L. saitoi* was previously described [11]. Most of the NMR signals for compounds **3** and **4 **were duplicated or overlapped. By detailed analysis of 1D and 2D NMR data, their structures were determined to be C-9 epimer of 6,9-epoxybisabola-2,7(14),10-triene.

The molecular formula of compounds **3** and **4** were determined to be C_15_H_22_O (five degrees of unsaturation) on the basis of HRESIMS data. Examination of the ^1^H and ^13^C NMR data (Table 2) revealed that they resembled 9*S*- and/or 9*R*-2-bromo-3-chloro-6,9-epoxybisabola-7(14),10-diene [11], except for the presence of signals for a trisubstituted vinyl group at C-2 and, accordingly, the lack of the resonances due to a brominated methine at C-2 and a chlorinated quaternary carbon at C-3 [11]. The chemical shifts for the vinyl carbons at *δ*_C_ 119.1/119.0 (C-2) and 133.4/133.7 (C-3) as well as for one of the neighboring methylene groups C-4 (*δ*_C_ 27.7/28.0) in the ^13^C NMR spectrum of **3** and **4 **were very similar to those reported for 8-bromochamigra-1,11(12)-dien-9-ol (with C-2 at *δ*_C_ 119.4, C-3 at *δ*_C_ 132.9, and C-4 at *δ*_C_ 27.5) [12], and these data strongly supported the presence of the trisubstituted vinyl group at C-2 in **3**/**4**. These data indicated that compounds **3** and **4 **were the dehalogenated derivatives corresponding to 9*S*- and/or 9*R*-2-bromo-3-chloro-6,9-epoxybisabola-7(14),10-diene [11]. The ^1^H–^1^H COSY and HMBC correlations (Figure 2) further verified the planar structures of **3**/**4** to be 6,9-epoxybisabola-2,7(14),10-triene. Assignment of the relative configuration at C-6 by NOESY experiment is not applicable for compounds **3** and **4** since there is no proton around C-6 in the tetrahydrofuran ring. However, the C-6 relative configuration was tentatively assigned to be the same as that of 9*S*- and/or 9*R*-2-bromo-3-chloro-6,9-epoxybisabola-7(14),10-diene based on the similar NMR data around the chiral center, as well as on biogenetic consideration [11].

Okamurene E (**5**), a colorless oil, was shown to have the molecular formula of C_15_H_23_BrO by the interpretation of HRESIMS data. The IR absorption at 3401 cm^−1^ exhibited the presence of a hydroxyl group. The ^1^H NMR spectrum (Table 2) delineated four methyl singlets, one double doublet ascribable to an oxygenated/halogenated methine, and one multiplet and two doublets attributable to three olefinic protons. The ^13^C and DEPT NMR spectra (Table 2) displayed four methyls, three methylenes, four methines, and four quaternary carbons. Compared to the reported NMR data for 10-bromo-7α,8α-expoxychamigr-1-en-3-ol [12], compound **5** exhibited no resonances for the epoxy moiety in the NMR spectra. Instead, it showed additional signals at *δ*_H_ 5.23 (H-8) and *δ*_C_ 139.5 (C-7) and 120.8 (C-8) for a trisubstituted vinyl group, which was positioned at C-7 based on the observed HMBC correlations from H-14 to C-6, C-7, and C-8. Further analysis of the ^1^H–^1^H COSY and HMBC correlations (Figure 2) confirmed the structure of **5 **as 10-bromo-1,7-chamigradien-3-ol. The relative configurations at C-3, C-6, and C-10 of **5** were deduced to be same as those of 10-bromo-7α,8α-expoxychamigr-1-en-3-ol [12] by the NOESY correlation between H-5 and H-10 as well as by their similar NMR data.

**Table 2:** ^1^H- and ^13^C-NMR data of compounds **3**–**6** in CDCl_3_^a^.

No.	3		4		5		6	
	*δ*_H_ (*J* in Hz)	*δ*_C_	*δ*_H_ (*J* in Hz)	*δ*_C_	*δ*_H_ (*J* in Hz)	*δ*_C_	*δ*_H_ (*J* in Hz)	*δ*_C_
1	2.25, m	38.1, CH_2_	2.18, m	36.8, CH_2_	5.54, d	131.2, CH	9.80, br s	199.3, CH
					(10.4)			
2a	5.34, m	119.1, CH	5.34, m	119.0, CH	5.85, d	136.5, CH	2.67, dd	42.4, CH_2_
					(10.4)		(17.5, 6.2)	
2b							3.06, dd	
							(17.3, 7.9)	
3		133.4, C		133.7, C		67.4, C	4.34, t (6.5)	72.7, CH
4a	1.93, m	27.7, CH_2_	1.93, m	28.0, CH_2_	1.56, m	28.5, CH_2_	4.65, dd	81.6, CH
							(8.7, 5.0)	
4b	2.22, m		2.22, m		1.99, m			
5a	1.58, m	31.5, CH_2_	1.66, m	34.5, CH_2_	1.78, m	36.3, CH_2_	2.75, m	21.7, CH_2_
5b	1.82, m		1.75, m				2.91, m	
6		80.9, C		80.8, C		47.4, C	4.97, m	80.9, CH
7		156.3, C		156.5, C		139.5, C	4.21, m	50.4, CH
8a	2.38, m	40.1, CH_2_	2.38, m	40.3, CH_2_	5.23, m	120.8, CH	2.42, dd	41.7, CH_2_
							(14.1, 5.8)	
8b	2.71, dd (15.7, 9.7)		2.61, dd (15.6, 9.5)				2.61, m	
9	4.63, m	71.8, CH	4.63, m	72.8, CH	2.58, m	36.1, CH_2_	4.50, dd	74.4, CH
							(7.4, 3.5)	
10	5.22, m	126.0, CH	5.22, m	126.2, CH	4.64, dd	61.4, CH	3.80, dt	64.1, CH
					(10.6, 6.4)		(11.5, 3.5)	
11a		136.2, C		135.5, C		41.6, C	1.77, m	27.4, CH_2_
11b							1.88, m	
12	1.69, s	18.2, CH_3_	1.70, s	18.3, CH_3_	1.02, s	18.1, CH_3_	1.07, t (7.7)	12.8, CH_3_
13	1.71, s	25.8, CH_3_	1.71, s	25.8, CH_3_	1.11, s	26.3, CH_3_		
14a	4.78, br s	103.5, CH_2_	4.78, br s	103.8, CH_2_	1.57, s	21.9, CH_3_		
14b	4.90, br s		4.91, br s					
15	1.66, s	23.4, CH_3_	1.66, s	23.4, CH_3_	1.31, s	28.8, CH_3_		

^a^ Measured at 500 MHz for ^1^H and 125 MHz for ^13^C.

Okamuragenin (**6**), isolated as a colorless oil, was assigned the molecular formula C_12_H_18_Br_2_O_3_ on the basis of HRESIMS, consistent with three degrees of unsaturation. The IR spectrum exhibited strong absorptions at 2762 and 1728 cm^−1^, indicating the existence of an aldehyde group. In accordance with the IR signals, the ^1^H and ^13^C NMR data (Table 2) also indicated the presence of an aldehyde group at *δ*_H_ (9.80, H-1) and *δ*_C_ 199.3 (CH, C-1). The ^1^H–^1^H COSY spectrum revealed that the aldehyde group was extended to a straight spin system consisting of six methines, four methylenes, and terminated by a methyl group (Figure 2). Compound **6** was deduced to be bicyclic, since no other unsaturated functionalities were indicated by the NMR data (Table 2). The connectivity of C-3/O/C-9 was deduced by the correlation from H-3 to C-9 in the HMBC spectrum (Figure 2). Taking into account the downfield chemical shifts of C-4 (*δ*_C_ 81.6) and C-6 (*δ*_C_ 80.9) and the calculated 3 degrees of unsaturation, C-4 and C-6 had to be linked through an oxygen atom. Finally, the two remaining Br-atoms indicated by the molecular formula could only be located at C-7 and C-10 based on the chemical shifts [13]. The relative configuration was determined by NOESY experiment. The same orientation of CH_2_-2, H-4, and H-9 was evidenced by the NOE correlations of H-2 to H-4 and H-9, while H-9 was *syn* to H-7 based on the NOE correlation between them. The above data established the structure of **6**, trivially named okamuragenin.

In addition to the six new compounds, the other nine sesquiterpenes including isobromocuparene [14], 7-hydroxylaurene [15], laurene [16], filiformin [17], debromofiliformin [18], 6-bromo-filiformin [19], deoxyprepacifenol [20], 2-bromo-3-chloro-2,7-epoxy-9-chamigren-8α-ol [11], and 2,10-dibromo-3-chloro-7-chamigren-9-ol [21], together with four C_15_-acetogenins including 3*E*, 12*Z*-laurediol [22], neolaurallene [23], *E*-stereoisomer of neoisoprelaurefucin [24], and 3*Z*-laurentin [25], were all identified by comparison of their spectral data with those previously reported.

The isolated compounds were evaluated for the brine shrimp (*Artemia salina*) lethal activity [26,27]. Among them, 7-hydroxylaurene was found to possess potent lethality with LD_50_ 1.8 μM, which is more active than that of 7-hydroxylaurene acetate, allolaurinterol acetate, and laurene [12]. Analysis of structure-activity relationship showed that the 7-hydroxyl group in laurene sesquiterpenes may play a key role in the brine shrimp toxicity, and the activity reduced significantly after acetylation. The above data suggested that 7-hydroxylaurene may be a potent chemical defensive agent with cytotoxicity, although the hatchability test was not performed [27]. The other tested compounds only displayed moderate or weak activity (data not shown).

## 3. Experimental Section

### 3.1. General

IR spectra were measured on a Nicolet NEXUS 470 FT-IR spectrophotometer. Optical rotations were recorded on an Atago Polax-L polarimeter. UV spectra were determined on a Spectrumlab 54 UV-visible spectrophotometer. HRESIMS were run on a VG Autospec 3000 mass spectrometer. 1D and 2D NMR spectra were obtained at 500 and 125 MHz for ^1^H and ^13^C, respectively, on a Bruker Advance 500 MHz NMR spectrometer in CDCl_3_ with TMS as internal standard. Column chromatography (CC) was performed on Si gel (200–300 mesh, Qingdao Haiyang Chemical Co., Qingdao, China) and Sephadex LH-20 (Sigma). TLC was carried out with precoated Si gel plates (GF-254, Qingdao Haiyang Chemical Co., Qingdao, China).

### 3.2. Algal Material

The marine red alga *Laurencia okamurai* Yamada was collected along Weihai coastline in Shandong Province, China, in May, 2007, and was identified by B.-M. Xia, Institute of Oceanology, Chinese Academy of Sciences (IOCAS). A voucher specimen (HZ0705) has been deposited at the Key Laboratory of Experimental Marine Biology of IOCAS.

### 3.3. Extraction and Isolation

The dried and powdered alga *L. okamurai* (3.8 kg) was extracted with a mixture of CHCl_3_ and MeOH (1:1, v/v). The concentrated extracts were partitioned between H_2_O and EtOAc. The EtOAc-soluble fraction was loaded to Si gel column, eluting with a step gradient of increasing EtOAc (0%–100%) in petroleum ether (PE) to give eight fractions I–VIII. Fraction II eluted with PE/EtOAc 100:1 and was further purified by preparative TLC to afford a mixture of **3** and **4 **(5.6 mg). Fraction IV eluted with PE/acetone 100:1 and was further separated by preparative TLC to afford **1** (3.7 mg), **2** (4.7 mg), **6** (13.1 mg). Fraction VI eluted with PE/acetone 30:1 and was further separated by Sephadex LH-20 (MeOH) CC and preparative TLC to afford **5** (10.7 mg).

### 3.4. Computational Details

*Okamurene *A (**1**): Colorless oil; [α]^18^_D_ +2.3 (*c* 0.11, MeOH); UV (MeOH) λ_max_ (log ε) 221 (3.56) nm; IR (KBr) ν_max_ 3065, 2964, 2857, 1514, 1479, and 1205 cm^−1^; ^1^H and ^13^C NMR data, see Table 1; HRESIMS *m/z* 297.0748 [M + H]^+^ (calcd for C_15_H_22_^79^BrO, 297.0854).

*Okamurene *B (**2**): Colorless oil; [α]^18^_D_ +3.6 (*c* 0.06, MeOH); UV (MeOH) λ_max_ (log ε) 221 (3.66) nm; IR (KBr) ν_max_ 3068, 2964, 2857, 1514, 1477, and 1208 cm^−1^; ^1^H and ^13^C NMR data, see Table 1; HRESIMS *m/z* 319.0726 [M + Na]^+^ (calcd for C_15_H_21_BrONa, 319.0673).

*Okamurenes *C (**3**) and D (**4**): Colorless oil; IR (KBr) *ν*_max_ 3096, 2924, 2854, 1637, 1457, and 1024 cm^−1^; ^1^H and ^13^C NMR data, see Table 2; HRESIMS *m/z* 219.1757 [M + H]^+^ (calcd for C_15_H_23_O, 219.1749).

*Okamurene *E (**5**): Colorless oil; [α]^18^_D_ +7.6 (*c* 0.09, MeOH); IR (KBr) ν_max_ 3401, 2971, 2928, 1549, 1447, 1367 and 1121 cm^−1^; ^1^H and ^13^C NMR data, see Table 2; HRESIMS *m/z* 281.0846 [M − H_2_O + H]^+^ (calcd for C_15_H_22_^79^Br, 281.0905), and 283.0860 [M − H_2_O + H]^+^ (calcd for C_15_H_22_^81^Br, 283.0884).

*Okamuragenin *(**6**): Colorless oil; [α]^18^_D_ +11.2 (*c* 0.18, MeOH); IR (KBr) ν_max_ 3060, 2926, 2854, 2762, 1728, 1421, and 1134 cm^−1^; ^1^H and ^13^C NMR data, see Table 2; HRESIMS *m/z* 385.9926 [M + NH_4_]^+^ (calcd for C_12_H_22_N^79^Br_2_O_3_, 385.9966), 387.9986 [M + NH_4_]^+^ (calcd for C_12_H_22_N^79^Br^81^BrO_3_, 387.9946).

### 3.5. Brine Shrimp Toxicity

Brine shrimp (*Artemia salina*) toxicity of crude extract and pure compounds was determined as detailed previously [26,27].

## 4. Conclusions

Four new bisabolane sesquiterpenes, okamurenes A–D (**1**–**4**), a new chamigrane derivative, okamurene E (**5**), and a new C_12_-acetogenin, okamuragenin (**6**), together with 13 known related metabolites, were isolated from the marine red alga *L. okamurai*. Among them, okamurenes A and B (**1** and **2**) are first examples of bromobisabolane sesquiterpenes possessing a phenyl moiety among *Laurencia*-derived sesquiterpenes, while okamuragenin (**6**) was the first acetogenin aldehyde possessing a C_12_-carbon skeleton. Each of the isolated compounds was evaluated for the brine shrimp (*Artemia salina*) lethal assay and 7-hydroxylaurene displayed potent lethality with LD_50_ 1.8 μM.
